# Books and Pamphlets Received

**Published:** 1892-08

**Authors:** 


### Books and Pamphlets Received.

The Operative Treatment of Goitre, by J. Collins Warren, M. D., Boston, Mass.

Biographical Sketch of John C. Hupp, A. M., M. D., of Wheeling, W. Va.

Transactions of the American Dermatological Association, at its fifteenth annual meeting, held at the Shoreham Hotel, Washington, D. C., on the 22d, 23d, 24th and 25th of September, 1891.

The Treatment of Tuberculosis of the Bones and Joints by Parenchematous and Intra-articular Injections, by N. Senn, M. D., Ph. D., Chicago, Ill.

The Effect of Fluids on the Strength of Catgut, by D. Braden Kyle, M. D., Philadelphia.

Otitis Media—Abscess of Neck—Death, by E. Oliver Belt, M. D., Washington, D. C.

The Mission of the Association of Military Surgeons of the National Guard of the United States, by N. Senn, M. D., Ph. D., Chicago, Ill.

One Hundred and Fifty Circumcisions and the Lessons they Teach, by B. Merrill Ricketts, M. D., Cincinnati.

Hepatic Abscess—Report of a Case, with Remarks upon the Amœba Coli, by William A. Edwards, M. D., and James Sears Waterman, M. D., San Diego, Cal.

An Operating Table for General and Gynecological Surgery, adapted to give the Wendelenburg Posture, by Clement Cleveland, M. D., New York City.

Proceedings of the Florida Medical Association, Session 1892.

Report of Twelve Cases of Herniotomy, by B. M. Ricketts, M. D., Cincinnati, O.

What to do in Case of Accident, by B. Merrill Ricketts, Ph. D., M. D., Cincinnati, O.

Essentials of Medical Diagnosis, by Cohen and Eshner. W. B. Saunders, Philadelphia.

A New Pronouncing Dictionary of Medicine, by John M. Keating, M. D., L. L. D., Philadelphia. W. B. Saunders, Philadelphia.

